# Views on dementia among informal caregivers of people with dementia: A scoping review and thematic analysis of qualitative studies

**DOI:** 10.1177/13872877261420210

**Published:** 2026-03-03

**Authors:** Lina Charlotte Jeran, Anne Blawert, Anna Grünewald, Swen Staack, Anna Jannes, Jochen René Thyrian

**Affiliations:** 1 581365 Interventional Health Care Research, German Center for Neurodegenerative Diseases, site Rostock-Greifswald, Greifswald, Germany; 2 Kompetenzzentrum Demenz in Schleswig-Holstein, Norderstedt, Germany; 3 Institute for Community Medicine, University Medicine Greifswald, Greifswald, Germany

**Keywords:** aging, Alzheimer's disease, caregiving, dementia

## Abstract

**Background:**

Alzheimer's disease is common in later life and affects the person with dementia as well as their family. As the disease progresses, declining functions of activities of daily living increase dependence on relatives for support, who can become caregivers.

**Objective:**

To summarize the current state of knowledge regarding caregivers’ views on Alzheimer's disease and other types of dementia, and to identify overarching themes.

**Methods:**

We conducted a scoping review using PRISMA guidelines. Inclusion criteria were: a) qualitative studies or qualitative sections of mixed-methods studies about views on dementia among informal caregivers, b) publication between 2013 and 2023, c) publication in a peer-reviewed journal, d) English or German language. The search was carried out in five scientific databases (MEDLINE, PsycInfo, PSYNDEX, CINHAL, Web of Science). Information on authors, years, settings, participants, aims, methods, type of analysis, and results were extracted. Using reflexive thematic analysis, themes of views on dementia reported in the given articles were summarized.

**Results:**

We identified 42 relevant studies reporting views on dementia in informal caregivers and constructed seven themes: “Dementia as natural cognitive decline”, “Dementia as caregiver burden”, “Dementia as stigmatized experience”, “Dementia as transition in relationship dynamics”, “Dementia as uncertainty”, “Dementia as enriching experience” and “Dementia as self-inflicted vs. externally determined”.

**Conclusions:**

Views on dementia among informal caregivers encompass complex, multi-dimensional attitudes and perceptions warranting a nuanced dementia discourse and offering various starting points for interventions. “Dementia as transition in relationships dynamics” emerged as an especially important topic requiring more attention in dementia research.

## Introduction

### The need for an umbrella term: Views on dementia

As the number of dementia cases continues to rise,^1^ members of the general public are increasingly likely to encounter the disease and people living with it in their everyday lives. Accordingly, studies on how people understand and perceive dementia are gaining relevance. However, research on conceptions of dementia is scattered over many related constructs, such as research on individual illness perceptions regarding dementia, research on individual and societal attitudes and stigma around dementia, or dementia stereotypes. This inconsistency in terminology complicates the integration of knowledge on this topic. Thus, we suggest “views on dementia” as an umbrella term to consolidate the given constructs that have so far been researched rather isolated. Such an umbrella term is expected to be practical for organizing and structuring these various research directions. In this paper, we refer to the term “views on dementia” when describing findings on attitudes, perceptions, beliefs regarding dementia and people with dementia, as well as related constructs. Thereby, we draw on the definition on the widely recognized term “views on aging”.

### Dementia and views on aging

One of the most common misconceptions about dementia is the belief that dementia is a part of the normal aging process.^2^ This suggests that although dementia is a medical condition and not a direct result of old age, people's attitudes toward dementia and views on aging seem to intertwine. The umbrella term “views on aging” comprises individual and societal attitudes, beliefs, perceptions and stereotypes about age, aging and older adults.^3^ Views on aging are important predictors of health outcomes and even mortality.^4^ People often associate aging with decline and loss rather than gain, e.g., older people are viewed as “senile”, “sick” and “incompetent”.^5,6^ Similar adjectives might also arise when describing symptoms of dementia, i.e., significant cognitive decline that diminishes independence in daily activities.^7^ Thus, it could be argued that attitudes toward dementia represent an extreme form of negative views on old age. In other terms, developing dementia might be viewed as the manifestation of “unsuccessful aging”,^8^ running contrary to the widely used idea of “successful aging”^9^ as a developmental goal in later life. Yet, at its core, aging simply means the passing of lifetime with manifold associated changes, while dementia is a disease that not everyone will develop—thus, old age and dementia may not be equated. In accordance, the enduring stigma associated with dementia, including negative stereotypes, prejudice and discrimination is still a persistent global challenge, which requires sustained efforts to mitigate, as highlighted by recent WHO's global action plan^10^ and Alzheimer's Disease International World Alzheimer Report 2024.^11^

### Informal caregivers and their views on dementia

Family members and other informal caregivers provide a large portion of caregiving tasks for people with dementia: Global estimates indicate that in 2015, an equivalent of over 40 million full-time workers provided informal care for people with dementia.^12^ This number is expected to increase to 65 million workers by 2030. Thus, informal caregivers are crucial in reducing pressure on the health care system by delaying hospitalizations. Dementia is a progressive and terminal disease, i.e., as dementia symptoms become more severe people with dementia gradually lose their abilities to perform daily activities, eventually resulting in total dependence on others.^7^ As a consequence, caregivers have to take on more caregiving responsibilities over time. Hence, caring for someone with dementia can be challenging and caregivers often experience their situation as burdensome.^13–15^ Thus, there is a strong need to understand the perspective of informal caregivers on dementia in order to develop tailored support programs.

Due to their relationship and closeness to the person with dementia, informal caregivers differ from lay people in their views on dementia. Marhánková and Honelová^16^ discovered that both groups framed dementia as tragedy: Caregivers primarily viewed dementia as slowly losing a loved one and constructed dementia as a different way in which people approach the world (i.e., they were concerned with changes in the personality and perception of the person with dementia), while laypersons viewed dementia as loss of overall humanity.

### Illness representations as part of views on dementia

One important construct that has been studied in regards to views on dementia are illness representations of dementia as proposed by Leventhal's self-regulation model.^17,18^ The self-regulation model suggests that our beliefs about an illness (cognitive illness representations) and emotional responses to it, such as anxiety, depression or anger (emotional representations), influence how we cope with it. The self-regulation model identifies several key components of illness representations. These include: identity, timeline, causes, perceived controllability, illness coherence and consequences of the illness.^17,19^ Thereby, identity involves the disease label and perceived symptom characteristics. Timeline refers to the expected length of development and duration of the illness as well as whether it is acute, cyclical or chronic. Causes are the assumed contributing factors that led to the illness. Perceived controllability refers to one's beliefs about treatability and curability. Illness coherence is the feeling of understanding one's illness, and lastly, consequences are the expected short- and long-term effects of the illness.

So far, only few studies investigated illness representations of dementia in caregivers. As part of a larger scoping review, Shinan-Altman and Werner^20^ summarized the results of five studies about informal caregivers’ illness representations of dementia, and discovered that informal caregivers labeled memory decline and confusion with time and place as the main symptoms of dementia. Aging, lifestyle factors and biological inheritance were identified as root causes of dementia by the caregivers. Furthermore, some of the informal caregivers perceived dementia as having severe consequences.

Taken together, research on different facets of views on dementia has yielded valuable insights into how people construct and perceive the illness. To consolidate research on these facets and to move the study of views on dementia forward, we conducted a scoping review and an in-depth thematic analysis of current studies focusing on different facets of views on dementia in informal caregivers. It is reasonable to assume that studying views on dementia and its implications is an important step to foster the health and wellbeing of those affected by dementia and for people involved in informal and formal care.

## Methods

This scoping review is reported in accordance with the Preferred Reporting Items for Systematic Reviews and Meta-Analyses extension for Scoping Reviews, PRISMA-ScR.^21^ The corresponding protocol to this review is accessible on OSF via the following link: https://osf.io/jn4ms

### Eligibility criteria

Qualitative studies and qualitative sections of mixed-method studies about views on dementia among informal caregivers of people with different dementia diagnoses (Alzheimer's disease, Lewy body dementia, vascular dementia, etc.) and mild cognitive impairment were included. Further, there was a focus on recent studies published between 2013 and 2023. Studies about former informal caregivers who retrospectively told their stories were also included. Only peer-reviewed articles published in English or German, excluding book chapters, comments and dissertations, were taken into consideration.

### Information sources

The databases MEDLINE (via PubMed), PsycInfo (via EBSCO), PSYNDEX (via EBSCO), CINHAL (via EBSCO) and Web of Science (via Clarivate) were searched for relevant information.

### Search strategy

The search algorithm consisted of three strings, which were added with the “AND” operator. The strings included keywords and MeSH terms, synonyms, and truncations of words, which were combined with the “OR” operator. The first string contained the keywords “caregivers” and “family”. The second string included “dementia”, “Alzheimer's”, “mild cognitive impairment”, and the last string included “illness representations”, “attitudes toward dementia”, “stereotypes” and related constructs. A specific example of a complete search is provided in the protocol. The meta-information of this protocol entails adjustments for the different databases.

### Data management

EndNote (Version 20) was employed to store the selected papers and worked with the software “Covidence”^22^ to simplify the review process. Covidence was utilized to automatically and manually remove duplicates, screen papers (title/ abstract and full text), detect disagreements, extract information and export data, e.g., the PRISMA chart and interrater reliability coefficients.

### Selection process

Two of our review team members (LJ and AG) independently screened the papers for inclusion. A checklist regarding the criteria was provided and accessible via Covidence during the whole screening process. The screening process consisted of four stages: 1) pilot screening of titles/abstracts, 2) title/abstract screening, 3) pilot screening of full texts, and 4) full-text screening. The pilot screenings were training sessions where a small set of papers was evaluated and almost complete agreement regarding in/exclusion was achieved, resulting in a Cohen's κ > 0.81^23,24^ at the end of the session. This was required to ensure the quality of the title/abstract screening and full-text screening. Disagreements during screening were resolved through discussion. Cohen's κ was 0.47 for title/abstract screening and 0.42 for full-text screening, indicating moderate agreement both times.^24^

### Data extraction and synthesis

Two research group members (LJ and AG) independently extracted relevant data from the chosen articles. A template was employed to structure information on the author, year, setting, participants, aims, methods, analysis and summary of results of interest. We summarized the results regarding informal caregivers and views on dementia obtained via qualitative methods. Disagreements were solved through discussion.

### Data analysis

We applied thematic analysis (TA) “to find repeated patterns of meaning”^25^^(p.86)^ in the data. This method was preferable due to the characteristics of the data (big, heterogeneous) and the objective of this review to identify patterns in the studies and to focus on themes rather than detailed content.^26^ A reflexive TA with an inductive approach and a combination of codebook procedures was employed.^27^ The two researchers who had already conducted the screening/extraction process (LJ and AG) and were familiar with the material analyzed the papers, following the six steps for conducting thematic analysis presented by Braun and Clarke^25^: data familiarization, generating codes, creating themes, reviewing the themes, defining and naming themes and, lastly, writing the report. In contrast to classical TA, a codebook-like structure to organize the codes was utilized. We excerpted relevant passages of the papers in the form of paraphrases (∼10,000 words), then coded those and constructed themes. Multiple iterations of possible themes were generated, discussed and optimized.

## Results

**Figure 1.**
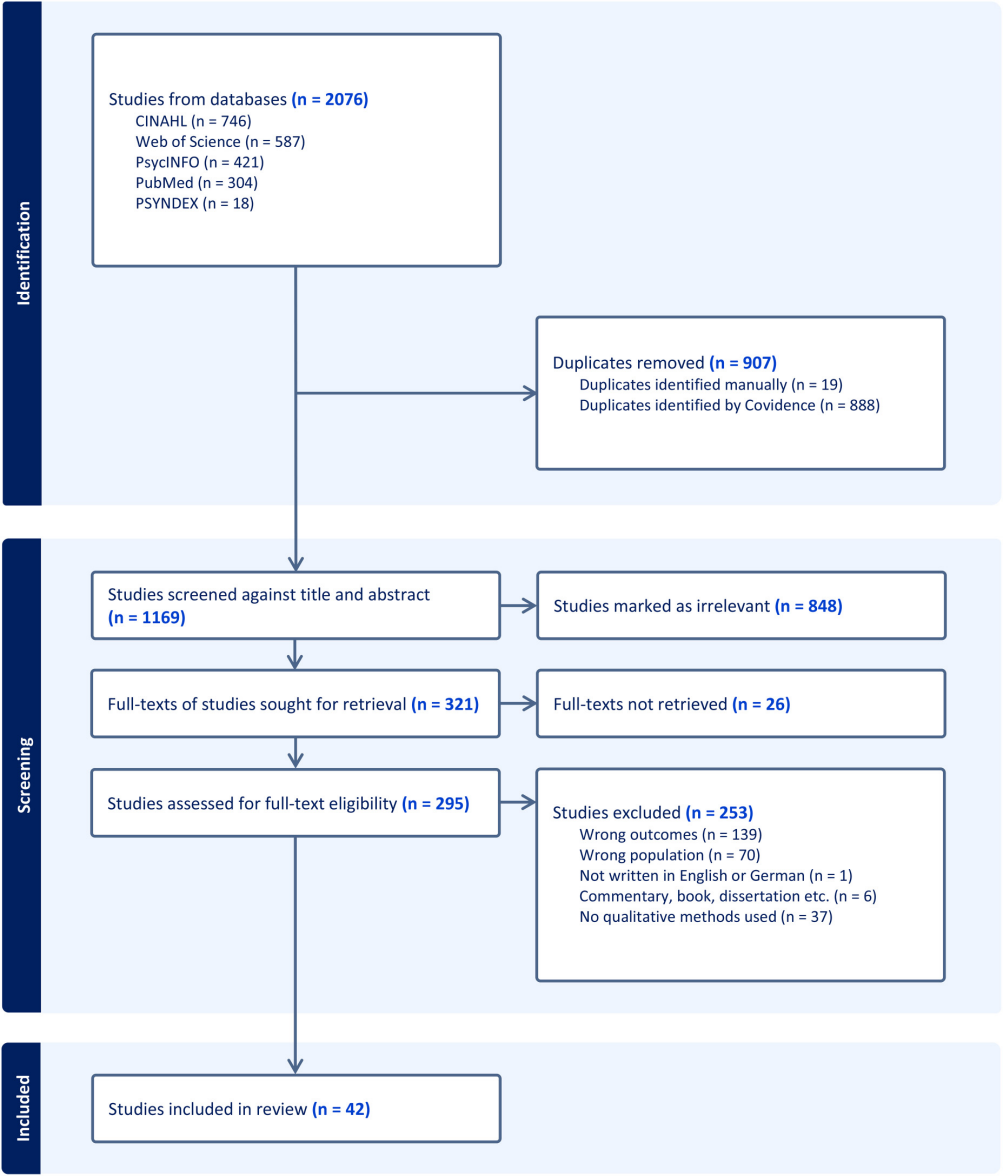
Flow chart of the study selection process.

The selection process with the number of included and excluded papers and our reasons for our decisions are presented in the PRISMA flowchart below (Figure 1). The study flow chart is reported in accordance with the PRISMA guidelines.^21^ We included 42 studies for data extraction and analysis (Table 1). The overall sample size consisted of 873 caregivers. Only 27 studies reported the mean age of their participants. Across those studies, the general mean age was 57 years. We included papers from 22 countries: Australia, Brazil, Canada, China, Germany, India, Iran, Israel, Kenya, Mexico, The Netherlands, Nigeria, Norway, Pakistan, South Africa, Spain, Sweden, Tanzania, Turkey, UK, USA, and Vietnam. With the exception of one mixed method study, all studies were qualitative studies. We constructed seven common themes comprising 17 subthemes in the included studies (Table 2).

**Table 1.** Review of the included papers about views on dementia among informal caregivers.

Source	Year	Country	Population	Method and analysis	Aim	Main results
Abojabel & Werner^28^	2019	Israel	20 Israeli Arab family caregivers (M = 54 years)	Focus groups and theory-led codebook thematic analysis	Understanding family stigma of Alzheimer's disease through experiences of Israeli-Arab caregivers (spouses and children)	Israeli Arab informal caregivers reported experiencing both public and affiliate family stigma with cognitive attributions (e.g., crazy, filthy, neglectful, punished by God), emotional (e.g., disgust, misunderstood, anger, fear, shame, pity) and behavioral reactions (e.g., isolation, concealment, avoiding help-seeking).
Agyeman et al.^29^	2019	UK	28 people, including 10 people with dementia and their family caregivers	Interviews, framework approach	Investigating the sociocultural beliefs, comprehension, perspectives and actions associated with living with dementia in Kintampo, Ghana	Dementia was seen as a cognitive decline, caused by stress, supernatural powers or age. Caregivers reported being burdened financially due to progression of the disease. Western and traditional medications were seen as plausible treatment options.
Allam et al.^30^	2023	Australia	14 people from the Australian Arabic-speaking community, including: 3 people aged over 70 with cognitive changes/ dementia symptoms, 6 caregivers (M = 48 years), 5 health care professionals	Semi-structured interviews and reflexive, data-driven thematic analysis	Exploring the perception of dementia symptoms, help-seeking and support in the given community	Allam et al. constructed seven themes. Memory loss, confusion and childlike behaviors were seen as symptoms of dementia and keeping routines/ making the person with dementia comfortable were reactions to those symptoms. Barriers to help-seeking were hiding dementia, cultural values/ family-oriented care, lack of knowledge. Facilitators for help-seeking were education of the community and culturally appropriate support.
Andrews et al.^31^	2017	Australia	10 family caregivers of people with dementia resident in a dementia specific unit in Australia	Semi-structured interview and iterative/thematic analysis	Investigating how family members of people with advanced dementia understand the condition	Most family members did not have knowledge on dementia being a terminal condition. The families reported a lack of knowledge and miscommunication with the care staff.
Ar & Karanci^32^	2019	Turkey	20 primary, adult-child caregivers of people with Alzheimer's disease from Turkey (M = 43.3 years)	Semi-structured interviews and interpretative phenomenological analysis	Understanding Turkish adult-child caregivers' perception of Alzheimer's disease and their caregiving experience	Ar & Karanci created four topics: causes of disease (mostly psychological stress), changes in the relationship between caregiver and people with dementia, people with dementia's personality, psychosocial and positive changes in caregivers, coping strategies (religion and social support) and (negative) appraisal of nursing home placement.
Araújo et al.^33^	2017	Brazil	5 family caregivers of people with Alzheimer's disease, residing in the municipality of Montes Claros (38–62 years)	Semi-structured interviews, thematic content analysis	Explaining the consequences of Alzheimer's disease on the lives of the family caregivers	Themes generated were: caregivers' (lack of) knowledge about Alzheimer's disease, feelings unveiled by the caregiver (sadness, fatigue, revolt etc.), caregiver's overload (Alzheimer's disease affects caregiver's daily life, leads to neglecting their own life), major difficulties encountered by the caregivers (caregiving without support from others is tiring), strategies used by the caregivers in coping with behavioral changes in patients with Alzheimer's disease (praying, listening to music etc.).
Balouch et al.^34^	2021	Pakistan	20 Pakistani family caregivers of people with dementia (M = 52.1)	Semi-structured interviews and reflexive, data-driven thematic analysis	Exploring the knowledge about symptoms, responses to the symptoms and access of help reported by Pakistani family caregivers of people with dementia.	Balouch et al. created five themes: knowledge and awareness (lack of awareness), stigma (mostly positive community reactions), importance of religion and duty to care, use of day care centers and home support and barriers.
Bjørge et al.^35^	2019	Norway	15 family caregivers (39–92 years) in Norway	Semi-structured interviews and systematic text condensation	Understanding caregivers' experience on how they perceived relationship changes during the course of dementia	Bjørge et al. constructed an overall theme experience of companionship with the sub themes experience of loss and loneliness, experience of communication and deterioration, experience of role change and experience of caring considerations and coping resources.
Dai et al.^36^	2013	China	13 Chinese family caregivers of people with mild cognitive impairment (M = 68.5 years)	Semi-structured interviews, grounded theory	Examining the experience and knowledge of mild cognitive impairment among the given population	Dai et al. identified three themes: initial recognition of cognitive decline (initial diagnosis only after encounter with specific triggers), experience of the diagnosis (unsatisfied with treatments, trying to keep a routine, worry, stigma), perception of cognitive decline as a normal part of aging.
Dai et al.^37^	2015	China	46 family caregivers of individuals with cognitive impairment in Wuhan and Beijing (41–85 years)	Semi-structured interviews, grounded theory	Studying the perception of mild cognitive impairment I/ Alzheimer's disease and caregiving among family caregivers in China	Dai et al. constructed two themes: Alzheimer's disease is natural (Alzheimer's disease involves discrimination, it is simply that way), family caregiving is a Chinese tradition (respecting the person with dementia as before, caring for family members, experiencing burden; practically no burden).
Feldman et al.^38^	2017	UK	84 family caregivers of people with dementia (M = 66 years)	Semi-structured interviews, Pearson's Chi-squared test, odds ratios	Exploring family caregivers’ perceptions of early signs/ onset of dementia and symptoms attribution	Caregivers described dementia as behavioral changes with memory decline, resulting from traumatic experiences in people with dementia's life, but also being a part of natural aging or others. In over a third help seeking was delayed by attribution of symptoms.
Gregorio et al.^39^	2015	Canada	15 caregivers, 2 people with dementia, 37 health/ social service providers, 17 other community members from Northern Ontario	Individual and group interviews, interpretative, constructionist paradigm	Exploring the dementia journey through the perspectives of the four different population groups in Northern Ontario	Due to lack of informational support by healthcare providers there was a lack of awareness and understanding about the disease, for example dementia being a natural part of aging. Caregivers stated that long term care in a nursing home would be an option with progression of disease.
Hall & Sikes^40^	2017	UK	22 children and young people (6–31 years old, the majority between 16–24) with parents with dementia	Interviews and narrative auto/ biographical methodologies/ thematic approach	Investigating the inadmissible stories of young people with parents with dementia	The process of narrating dementia was difficult for caregivers. Taboo subjects were: Not liking the ill parent, other illnesses being preferred to dementia, difficult behaviors through dementia, the portrayals of dementia from people with no experience differ from the caregivers’ ones.
Hedman, Norberg & Hellström^41^	2019	Sweden	10 people with Alzheimer's disease and their family caregivers (M = 66.4)	Individual interviews, content analysis	Understanding perspectives on Alzheimer's disease by people with dementia and caregivers	Person with Alzheimer's disease rated their abilities higher than the caregivers did. Caregivers were reported to have a lack of knowledge, which impacted the sense of communion of the person with Alzheimer's disease.
Hellström & Torres^42^	2021	Sweden	22 spouses of people with dementia (M = 68.4)	Interviews, thematic analysis (data driven, no specific approach)	Investigating the perceptions of people without dementia on everyday competences of the people with dementia	Partners caring for their spouse with dementia mainly showed two different approaches to caregiving. Either using a couple centered approach and managing the situations together or being overwhelmed by the situation leading to stress, taking over tasks and diminishing people with dementia's everyday competences.
Hossain et al.^43^	2019	UK	6 Bangladeshi family caregivers living in London and Portsmouth (M = 44.16 years)	Semi-structured interviews, synthesis by meta-aggregation (Joanna Briggs Institute)	Exploring the caregiver's daily experiences and knowledge	Hossain et al. constructed two broad themes: early symptoms and diagnosis of dementia and myths and stigma around dementia. There was a lack of knowledge and awareness of symptoms. Dementia was seen as a medical condition with no stigma attached.
Hurzuk et al.^44^	2022	India	8 Indian persons with dementia (M = 66.7 years), 19 family caregivers (M = 49.8 years), 16 health care professionals (M = 33.5), 15 people from the general public (M = 49.3 years)	Focus groups and individual interviews, codebook thematic analysis	Understanding attitudes towards people with dementia	Hurzuk et al. identified three themes: poor awareness (lack of knowledge and misconceptions), stigma (stigmatizing language, behaviors, beliefs) and barriers to accessing care (Treatment/ care pathways gaps, availability of specialist care, accessibility of care/ resources, cost and time). Overall, socio-cultural aspects like culture, religion and societal norms influenced the beliefs of the participants.
Juárez-Cedillo, Jarillo-Soto & Rosas-Carrasco^45^	2014	Mexico	8 primary caregivers of people with dementia (M = 55 years)	Semi-structured interviews, content analysis	Exploring family caregivers' experience with dementia and how the awareness of social representation impacts help-seeking	Caregivers reported being emotionally burdened by the role, due to lack of formal and informal support, leading to feelings of sadness and a lack of freedom in their own life. Dementia was described as a disease of the old, leading to craziness, which was associated with stigma.
Lee Casado et al.^46^	2015	USA	23 Korean American family caregivers of people with dementia (M = 67.3 years)	Focus group interviews and codebook, thematic analysis with a team consensus approach	Studying the experience of dementia caregiving among Korean Americans	Family caregiving was presented as entailing numerous burdens, such as language barriers, negative feelings about caregiving experience, and a cultural obligation to look after the person with dementia.
Mahomed & Pretorius^47^	2022	South Africa	30 Black South African family caregivers of people with dementia (M = 45.6 years)	Semi-structured interviews, reflexive, data-driven thematic analysis	Investigating South African family caregivers’ experiences	The themes Mahomed & Pretorius constructed were: understanding dementia (spiritual problem, forgetting due to ageig, medical illness, going mad, challenging, lack of awareness), struggles and sacrifice (personal sacrifices, role changes, lack of support, withdrawal), mental health (psychological distress, grief) and protective factors (commitment, coping, personal growth etc.).
McDarby et al.^48^	2023	USA	40 current or recently bereaved family caregivers (M = 70 years) of home hospice patients living with dementia	Semi-structured interviews, codebook thematic analysis (data driven /deductive)	Identifying caregiving challenges and strategies of caregivers of people with dementia	The study focused on end-of-life informal caregiving providers. These caregivers reported a lack of knowledge regarding the disease, receiving help from health care providers and accepting this help being associated with stigma. They reported it being hard for them to let go of the situation and stopping themselves from feeling guilty after organizing hospice care for the person with dementia.
Megido et al.^49^	2023	Spain	25 grandchildren living in Catalonia and being caregivers to people with dementia (6–13 years)	Semi-structured interviews, thematic content analysis	Studying the changes of the relationship between grandparents and grandchildren due to Alzheimer's disease	The grandchildren of people with dementia in the given community stated that they experienced a role reversal with their grandparents, helping with everyday tasks and adapting shared activities to people with dementia's level of capability. They described a variety of different experiences, the positive ones overweighing fears and worries. Themes created: Current Relationship, Grandchild as caregiver, current consequences of the disease.
Mogar & von Kutzleben^50^	2015	Germany	7 Turkish migrant family caregivers of people with dementia (M = 32.3 years)	Semi-structured interviews, grounded theory	Exploring the care of Turkish migrants with dementia in the home environment	Caregivers in the given community reported on lacking knowledge regarding dementia, which lead to barrier in help-seeking. As it was seen as a cultural necessity to look after ill members of the family, accepting caregiving facilities as nursing homes was seen as a form of betrayal. Within the family and community there reported a strong support network, which helped fulfilling the role.
Mohabbat Bahar & Bigdeli^51^	2020	Iran	15 Iranian people with dementia and their family caregivers (M = 48–65 years)	Semi-structured interviews, descriptive phenomenological method	Exploring stigma towards dementia in Iranian population	Two themes were identified: Dysfunctional beliefs (anxiety of deteriorating changes, anxiety of developing dementia oneself) and negative social attitudes (limited interactions, shame, incompetence).
Mushi et al.^52^	2014	Tanzania	25 people with dementia and 16 caregivers (19–58 years) of people with dementia from the Hai District of Kilimanjaro	In-depth interviews, content analysis	Exploring the beliefs regarding dementia and experiences of people with dementia and their caregivers in Tanzania (Hai District of Kilimanjaro)	Caregivers observed memory problems, but they were viewed as normal part of aging. Most caregivers did not know dementia or could state causes for development of dementia. Causes mentioned were other diseases, witchcraft, stress, old age. Modern and traditional medicine were acknowledged as treatment options. Caregiver burden was mentioned.
Musyimi et al.^53^	2021	Kenya	14 healthcare workers (M = 34.8 years), 17 people from the general public (M = 41.7 years), 12 family caregivers (M = 50.8 years)	Focus group discussion and five individual interviews with caregivers and reflexive, data- driven thematic analysis	Examining perceptions of dementia and dementia care in the three stakeholder groups in rural Kenya	Musyimi et al. created four themes: negatives stereotypes of dementia (dementia = “thinking high”, being cursed, part of normal aging), limited knowledge about dementia, diagnostic pathway (negative experiences, poor communication) and neglect and abuse.
Mwendwa et al.^54^	2021	Kenya	8 family caregivers and 2 paid caregivers (40–69 years) of people with dementia in Kenya	Semi-structured interviews, interpretative phenomenological analysis	Understanding the views/ experiences of being a caregiver for a person with dementia in Kenya	Themes identified were: Personal experience of caregiving, supports to assist with caregiving and the perceived unmet care needs. The experiences of the caregivers were influenced by (lack of) support, years of caregiving and (lack of) knowledge.
Nguyen et al.^55^	2020	USA	20 Vietnamese American family caregivers (M = 61.5 years) of people with Alzheimer's disease	Semi-structured qualitative interview, codebook thematic analysis	Studying the understanding of Alzheimer's disease and caregiving of family members in Vietnamese American population	Nguyen et al. constructed five themes: “Now I know:” the disruptions, shocks and surprises leading up to the initial diagnosis; The frustrations of managing family members’ cognitive impairments; “Going with the flow:” challenges in managing personality and behavioral changes; The exhaustion of around-the-clock caregiving; “Taking it day by day” in the face of progressively worsening symptoms.”
Nguyen et al.^56^	2021	Vietnam	12 family caregivers (M = 56 years), 9 healthcare providers	Semi-structured interviews, codebook thematic analysis	Investigating the experiences of family caregivers of people with dementia in Vietnam from the perspective of caregivers and health care professionals	Four themes were created: Perceptions of dementia as normal part of aging, family caregiving as moral obligation, gender and birth order in family caregiving and difficulties and challenges of family caregiving (e.g., losing time, loss of income, isolation, impaired physical health, emotional distress).
Nwakasi, de Medeiros & Bosun-Arije^57^	2021	Nigeria	12 adult informal caregivers in Nigeria (M = 48.9 years)	Semi-structured telephone interviews with caregivers and focus groups, qualitative descriptive approach	Exploring the perspectives on dementia and the impact of stigma	Themes generated were: Misconceptions about dementia symptoms (witchcraft, madness, normal aging), caregiving protects against stigmatization and stigma affects caregiving support (avoidance, poor support). There was an overall lack of knowledge and awareness.
Oliveira et al.^58^	2023	Brazil	6 people living with dementia and 15 family caregivers (M = 58 years)	Semi-structured interviews, critical narrative inquiry	Examining perceptions of people with dementia and family carers that are stigma-related in Brazil	Two themes for people with dementia were created: Managing negative views on dementia and negative experiences relating when interacting with others. Four themes for caregivers were created: Views and beliefs about dementia and people with dementia, power dynamics within caring relationships, exclusion and discrimination towards carers and people with dementia, essential components of caring
Parial et al.^59^	2023	China	38 older people (M = 74 years) and family caregivers (M = 54.8 years) from India, Pakistan, and Nepal who live in Hong Kong	Semi-structured interviews, reflexive, data-driven thematic analysis	Studying the knowledge, beliefs, and help-seeking behaviors of South Asian migrants in Hong Kong about dementia, as well as to identify barriers to obtaining dementia knowledge and help-seeking	Themes identified were: Normalization of stigmatization of dementia, spiritual and psychosocial attributions of dementia, familial responsibility despite potential caregiving burden, uncertainties versus openness to professional care and barrier and opportunities in dementia literacy.
Quinn, Jones & Clare^60^	2017	UK	50 informal caregivers of people with dementia (M = 66.5 years)	Semi-structured interviews, content analysis	Understanding (components of) illness representations of dementia in caregivers of persons with dementia	Caregivers employed medical labels and used Alzheimer's disease and dementia interchangeably. Biological causes, genetics, age, life events and environmental factors were seen as reasons for developing dementia, only few identified treatment options. The unpredictable future leaves the caregivers with feelings of being stressed and overwhelmed.
Richardson et al.^61^	2019	USA	15 informal caregivers of people with dementia (M = 62.7 years)	In-depth interviews, a modified thematic analysis (data-driven, reporting coding reliability)	Investigating the influence of culture on caregivers’ experiences and utilization of support services	The themes constructed were: Knowledge about dementia, language barriers, religion and spirituality and cultural differences in attitudes about caring and formal servers. They developed a model for intervention based on these themes.
Sagbakken, Spilker & Ingebretsen^62^	2020	Norway	12 family caregivers of people with dementia (25–78 years), 18 health personnel, 51 older immigrants	Focus groups, three different contexts of interpretation (self-understanding, critical common-sense understanding, theoretical understanding)	Exploring the understanding and view on dementia among relatives of immigrated people with dementia and others	Dementia was seen as a natural part of aging, leading to loss of abilities and personality changes of people with dementia. The behaviors of people with dementia were highly stigmatized leading to feelings of shame with the caregiver.
Santos et al.^63^	2013	Brazil	18 Latin American family caregivers of people with dementia (M = 78.09 years)	Interpretative phenomenological analysis	Investigating the differences in dementia awareness in Latin American caregivers who are participants of a psychoeducational group	Reasons to become a caregiver were duty, feelings of gratitude, familism, religion. There were difficulties in recognizing cognitive and functional impairments. They tried maintaining the person with dementia's past identity.
Strier & Werner^64^	2016	Israel	10 People with Alzheimer's disease, 25 family caregivers of people with Alzheimer's disease, 15 healthcare professionals in Israel	10 in-depth personal interviews, 4 focus groups, grounded theory	Exploring welfare stigma in long-term care insurance (LTCI)	Though people with dementia had stigmatic self-images, relatives and health care professionals had no stigmatic views regarding people with dementia or LTCI. Yet, eligibility determination procedures were experienced as being stigmatizing.
Tatangelo et al.^65^	2018	Australia	17 adult child primary caregivers (M = 51.1 years) of people with dementia in Australia	Semi-structured interviews and thematic content analysis	Understanding the experience of adult child caregivers	Themes created were: Family expectations and caregivers' lack of choice in adopting the caregiving role, denial and differential understandings of dementia among family members, differential beliefs and approaches to caregiving among family members and communication break down between family members.
Van Wezel et al.^66^	2018	Netherlands	69 Turkish, Moroccan, Surinamese Creole family caregivers living in the Netherlands (20–84 years)	Individual interviews, focus group interviews, generic qualitative approach	Studying the explanations of the given caregivers and openness about dementia viewed by female caregivers in the community/ family	Caregivers and people with dementia experienced stigma from the community, while within the family it was easier to talk about the disease without feelings of shame. They named various risk factors for dementia like psychological factors, traumatic life events but also spiritual disparities. Dementia was often seen as normal part of aging.
Vrijsen et al.^67^	2021	Netherlands	15 descendants of people with dementia (M = 48.8 years)	Focus group discussions, content analysis	Investigating the beliefs, knowledge, attitudes towards dementia in descendants of people with dementia	Descendants identified modifiable risk factors (poor diet, lack of cognitive activities, other illnesses, etc.). Nevertheless, they were uncertain about reducing their dementia risk. After sharing their experiences and knowledge, they were more eager to engage in risk reduction.
Werner, Shpigelman & Turgeman^68^	2020	Israel	6 family caregivers of people with dementia (M = 61.3 years), 10 healthcare professionals (M = 52.7 years)	Focus groups, codebook thematic analysis	Examining stigmatic experiences of family caregivers of people with early-onset dementia	Both family and professional caregivers experienced courtesy, structural stigma and negative emotions. Additionally, family members were confronted with public stigma. Antecedents of stigma were looking for a formal diagnosis and misconceptions of dementia. Consequences were emotional and financial burden.
Yahalom^69^	2019	Mexico	22 family caregivers from Teotitlán del Valle	Interviews, analysis guided by Nichter's formulation of idioms of distress	Studying caregivers’ perception of progressive memory loss	Caregivers perceived dementia as normal, also due to social constructs. There were many descriptions of trying to make sense of dementia, e.g., stress being a risk factor or talking about “soul loss”.

**Table 2.** Themes of the included papers about views on dementia among informal caregivers.

Theme	Subthemes
Dementia as natural cognitive decline	Dementia impairs cognitive abilities (n = 25)
	Dementia as part of natural aging (n = 21)
	Dementia can affect independence (n = 10)
Dementia as caregiver burden	Physical and psychological burden (n = 20)
	Economic burden (n = 11)
Dementia as stigmatized experience	Dementia can carry stigma (n = 15)
	Caregiving can be lonely and isolating (n = 17)
Dementia as transition in relationship dynamics	The caregiver can feel responsible for the person with dementia (n = 19)
	Shift in roles (n = 16)
	Dementia can change the personality of the person with dementia (n = 23)
	Dementia as tragedy (n = 21)
Dementia as uncertainty	Lack of knowledge and awareness (n = 16)
	Feelings of uncertainty (n = 16)
Dementia as enriching experience	Valuing the person with dementia (n = 13)
	Employing available resources (n = 15)
Dementia as self-inflicted vs. externally determined	Dementia as biological/ medical phenomenon (n = 15)
	Dementia as behavioral/ psychological phenomenon (n = 20)
	Dementia as supernatural phenomenon (n = 17)

### “Dementia as natural cognitive decline”

First, the theme “Dementia as natural cognitive decline” entails the three subthemes “Dementia impairs cognitive abilities”, “Dementia as part of natural aging” and “Dementia can affect independence”. This theme focuses on caregivers’ perceptions of the change in cognitive abilities and everyday functioning in people with dementia.

#### “Dementia impairs cognitive abilities”

Caregivers defined dementia mostly based on its cognitive symptoms, such as memory problems, forgetfulness, disorientation, confusion and impaired communication.^29,52,56^

#### “Dementia as part of natural aging”

Cognitive impairments were often viewed as part of the natural aging process, and caregivers had difficulties identifying the symptoms as belonging to an actual disease. Caregivers sometimes assessed dementia symptoms as harmless and ordinary: They downplayed the symptoms, saw them as relatable and overlooked the terminal nature of dementia.^31,42,59^

#### “Dementia can affect independence”

Some caregivers associated dementia with a loss of everyday competence and independence or described the person with dementia as “incapable”.^30,51,58^

### “Dementia as caregiver burden”

Second, we created the theme “Caregiver burden” with the subthemes “Physical and psychological burden” and “Economic burden”. It focuses on different kinds of caregiver burden.

#### “Physical and psychological burden”

Caregivers assisted people with dementia with multiple activities of daily living.^49,55,56^ As a consequence, caregivers experienced strain, which impacted their overall well-being, both emotionally and physically: Caregivers could feel uncomfortable or embarrassed when taking care of the personal needs of the person with dementia, for example their hygiene. Caregivers mentioned feeling overwhelmed, exhausted, anxious or stressed by caregiving.^33,47,56^ Caregivers reported sleeping and health problems.^32,46,49^ If the care recipient had had severe dementia, caregivers might had to constantly supervise the person with dementia and could not leave them alone. Thus, caregivers could feel trapped and experience a lack of personal freedom.^32,35,45^ To manage caregiving, caregivers may neglect their own needs.^33,46,48^ Caregivers might also have to handle changes in family dynamics, conflicts and tension.^32,49,65^

#### “Economic burden”

Economic burdens that were mentioned entailed financial burden, e.g., health care costs, loss of income, quitting one's job, borrowing money and loss of time due to the demands of caregiving.^29,47,68^

### “Dementia as stigmatized experience”

Third, we constructed the theme: “Dementia as stigmatized experience” with the subthemes: “Dementia can carry stigma” and “Caregiving can be lonely and isolating”.

#### “Dementia can carry stigma”

Some caregivers had to deal with stigma—not only did they observe the person with dementia being affected by stigma, but caregivers also faced affiliated stigma themselves.^28,57^ Observed stigma towards the person with dementia or toward the caregiver directly included: avoidance or not being visited anymore, talking behind ones back and being subjected to ridicule.^28,34,68^ It could also encompass physical abuse, the use of stigmatizing language and showing disgust or fear. One study discussed that caregivers would sometimes be perceived as crazy, filthy and neglectful by others.^28^ Caregivers might feel ashamed of the behavior of the person with dementia or themselves.^28,51,68^ The caregivers’ fear of stigma caused avoidance of talking about dementia outside of close family context, concealment of the disease and was a barrier to help seeking.^28,51,66^ Caregivers could feel the need to protect the person with dementia from stigma by hiding dementia symptoms from others.^51,57,68^ Caregivers occasionally reported that there was a negative association with the words “dementia” or “Alzheimer's disease” and that they tried to avoid it or replace it with another term.^36,53,60^

#### “Caregiving can be lonely and isolating”

Caregivers often mentioned a lack of formal or informal support.^47,54,57^ They could feel misunderstood by others or frustrated as they encountered situations where they were ignored, underappreciated, or their experiences were dismissed.^28,40,65^ Caregivers at times discussed being isolated from society, which was associated with feelings of loneliness.^28,44,53^ Stated reasons for seclusion were either being avoided due to stigma or self-isolation.

### “Dementia as transition in relationship dynamics”

The fourth theme “Dementia as transition in relationship dynamics” includes four subthemes: “The caregiver can feel responsible for the person with dementia”, “Shift in roles”, “Dementia can change the personality of the person with dementia” and “Dementia as tragedy”.

#### “The caregiver can feel responsible for the person with dementia”

In several studies, caregivers mentioned changing relationship dynamics in a way that they increasingly felt responsible for the person with dementia.^50,59,63^ This perceived obligation to take care of the person with dementia was attributed to moral (e.g., earning blessings from God), cultural (e.g., being a good wife or daughter) or familial (e.g., loyalty, commitment, giving back) values.

#### “Shift in roles”

Caregivers also mentioned the occurrence of a role change (becoming the protector and carer) or role-reversal, i.e., the person with dementia becomes the child while the caregiver plays the role of parent.^32,47,49^ People with dementia were characterized as “funny acting”, “child-like” or directly compared them to children or babies one needs to take care of and protect.^30,32,36^

#### “Dementia can change the personality of the person with dementia”

In several studies, caregivers reported changes in the personality of the person with dementia or that the person with dementia might even become a different person altogether.^35,54,55^ Sometimes, caregivers could not separate the personality of the person with dementia from the symptoms of the disease.^58,63^ Consequently, some caregivers reported disliking the person with dementia as they perceived them as being “manipulative”, “rude” or “selfish”.^40,53,58^ Furthermore, there were instances when caregivers interpreted dementia as a form of madness, i.e., the person with dementia was perceived as being “mad” or “strange”.^53,58,62^ Some caregivers reported aggressive behaviors or viewed the person with dementia as being “aggressive”, “difficult” or “angry”.^34,35,55^ Some caregivers described people with dementia as being absent, apathetic or emotionless.^31,35,64^

#### “Dementia as tragedy”

Caregivers often believed that dementia was a terrible, frightening disease that no one should have and framed it as a tragedy.^46,55,64^ Some caregivers believed that death or other illnesses, such as cancer, were preferable to dementia.^40,47^ Caregivers observed the symptoms progressively worsen over time in the person with dementia.^40,55,60^ Noticing the progression of the disease, caregivers could report the fear of no longer being recognized by the person with dementia and they felt sad when they were actually no longer recognized.^49,63^ The perceived loss of the person with dementia and the relationship with the person was mentioned by caregivers and portrayed as an especially painful experience, which was accompanied by sadness and grief.^32,35,54^ Caregivers also mentioned the fear of developing dementia themselves.^46,49,51^ Overall, there could be a sense of hopelessness and despair among caregivers when dealing with dementia.^54,55^

### “Dementia as uncertainty”

The fifth theme is “Dementia as uncertainty” with the subthemes “Lack of knowledge and awareness” and “Feelings of uncertainty”.

#### “Lack of knowledge and awareness”

Lack of knowledge about dementia became visible as in some studies caregivers either used a different term for dementia (e.g., “forgetfulness”) or had no label for the disease.^52,60,66^ If they knew about the term, they often used the terms “dementia” and “Alzheimer's disease” interchangeably.^31,46,60^ Caregivers often acknowledged that they had a lack of knowledge regarding dementia and wanted to receive or searched for further information themselves and continuously learned about dementia while caregiving.^31,46,48^ Lack of knowledge included limited understanding of the disease overall, the progression or symptom changes of dementia and how to manage the needs of the person with dementia. Caregivers’ lack of knowledge could often be attributed to miscommunication or lack of awareness by healthcare professionals. Studies found that sometimes caregivers had not heard of dementia before the person with dementia received their diagnosis, and they could be shocked by the diagnosis.^34,43,55^

#### “Feelings of uncertainty”

Further, caregivers were confronted with feelings of uncertainty or ambiguity concerning the disease and its progression; they were unsure of how to deal with unpredictable dementia behavior, and how to make sense of the disease.^29,47,60^

### “Dementia as enriching experience”

We call the sixth theme “Dementia as enriching experience”, which is about caregivers’ perception of positive aspects and acceptance of dementia. We assigned two subthemes: “Valuing the person with dementia” and “Employing available resources”.

#### “Valuing the person with dementia”

Caregivers often believed that people with dementia should be respected by others and one should make them feel comfortable and tend to their wishes.^36,37,62^ Some caregivers described the dementia journey as a bonding experience, improving or maintaining positive relationships with the person with dementia.^32,35,47^ Some also reported feeling close to the person with dementia as they shared more activities and spent time together.^35,58,64^ Caregivers talked about experiencing love, affection, compassion and empathy toward people with dementia.

#### “Employing available resources”

Caregivers perceived a variety of resources to handle the situation more easily. One important resource was informal support from family, friends and the community, as well as professional support from health care providers.^28,48,61^ To cope with the caregiving situation, other caregivers took joy in positive distracting and comforting activities, such as listening to music, reading or praying.^33,47^ Caregivers also experienced feelings of happiness and satisfaction while undertaking caregiving and being with the person with dementia.^32,35,49^ In some studies, caregivers observed positive personality changes in themselves, e.g., becoming more patient, understanding, resilient and overall growing as a person.^32,47,54^

### “Dementia as self-inflicted vs. externally determined”

The last theme, “Dementia as self-inflicted vs. externally determined,” includes caregivers’ different subjective theories of why dementia develops and how it can be treated. We created three subthemes to match this idea: “Dementia as biological/medical phenomenon”, “Dementia as behavioral/psychological phenomenon” and “Dementia as supernatural phenomenon”.

#### “Dementia as biological/medical phenomenon”

The subtheme “Dementia as biological/medical phenomenon” includes illness perceptions that dementia is a physical disease that is hereditary and caused by old age.^34,47,60^ Some caregivers noted that other illnesses, such as high blood pressure, stroke and diabetes, can be risk factors for the development of dementia.^34,52^

#### “Dementia is a behavioral/psychological problem”

“Dementia is a behavioral/psychological problem” encompasses the idea that dementia is a mental illness that is caused by lifestyle (drinking, smoking, etc.), stress and trauma, life events, overthinking and worry, loneliness and isolation or malnutrition.^66,67,69^ Social and cognitive stimulation are thought to help with the disease.^36,58,60^

#### “Dementia as supernatural phenomenon”

Finally, the subtheme *“*Dementia as supernatural phenomenon” involves illness perceptions that witchcraft and curses are the causes of dementia and that only traditional medicine, such as consultation by homeopathic doctors and traditional and transcendental healers, can treat the disease.^29,52,57^ Dementia is also often viewed as punishment from God, and caregiving is seen as a trial from God. Prayers and faith are employed to help with the situation.^28,52,66^

Overall, the 42 studies on informal caregivers’ views on dementia summarized in this scoping review were heterogenic in their methods and results. However, there were themes that emerged in several of these studies in a similar way. The most common were: “Dementia impairs cognitive abilities” (n = 25), “Dementia can change the personality of the person with dementia” (n = 23), “Dementia as tragedy” (n = 21) and “Dementia as part of natural aging” (n = 21).

## Discussion

The aim of this study was to identify, extract and analyze qualitative studies and qualitative sections of mixed-method studies on informal caregivers’ views on dementia. A total of 42 studies that addressed this topic could be identified. We created seven themes to summarize the findings of those studies: “Dementia as natural cognitive decline”, “Dementia as caregiver burden”, “Dementia as transition in relationship dynamics”, “Dementia as uncertainty”, “Dementia as stigmatized experience”, “Dementia as enriching experience” and “Dementia as self-inflicted vs. externally determined”. Most of the themes were in line with previous findings. One theme that seems to be of particular relevance regarding views on dementia among informal caregivers is “Dementia as transition in relationship dynamics”.

### Views on dementia and views on aging

Views on dementia and views on aging show several commonalities. Most prominently, the theme “Dementia as regression” and its subthemes match the stereotypical notion that old age is associated with decline and loss.^5,6^ Only when dementia symptoms became more severe, e.g., resulted in “abnormal” behavior, personality changes or loss of language, caregivers identified them as being different from normal consequences of aging.^55,62^ This might delay help seeking and fuel discrimination of people with young-onset dementia, as their illness is considered especially “unnatural”.^38,40,62^ However, caregivers also believed that people with dementia deserve respect, as older adults need to be honored.^36,37,62^

### Illness representation of dementia

The self-regulation model^17,18^ seems to be a general good fit to structure views on dementia, as all themes can be linked to components of the self-regulation model (Table 3). All seven components of the self-regulation model were mentioned by the caregivers in the studies. Regarding identity, the themes “Dementia as natural cognitive decline” and “Dementia as uncertainty” are relevant. Dementia is primarily labeled as cognitive decline.^29,52,56^ Alzheimer's disease and dementia are used interchangeably.^31,46,60^ Some caregivers do not have labels for the disease and use a substitute term or avoid labeling it at all.^52,60,66^ As taken up in the theme “Dementia as self-inflicted vs. externally determined”, caregivers state different causes (biological/medical, psychological/behavioral, and supernatural) and treatment options (Western vs. traditional medicine) for dementia.^29,52,59^ They also differ in their beliefs about the controllability of the disease, i.e., whether the disease can be treated or is incurable.^52,60,61^ Caregivers mostly believe that dementia symptoms worsen over time but fail to recognize dementia itself as a terminal illness and are unsure about the progression of the disease, as discussed in the themes “Dementia as uncertainty” and “Dementia as natural cognitive decline”.^29,31,55^

**Table 3.** Corresponding illness representation elements to the identified themes.

Theme	Illness representation
Dementia as natural cognitive decline	Identity, timeline
Dementia as caregiver burden	Consequences
Dementia as stigmatized experience	Consequences
Dementia as transition in relationship dynamics	Consequences
Dementia as uncertainty	Illness coherence, identity, timeline
Dementia as enriching experience	Consequences
Dementia as self-inflicted vs. externally determined	Causes, perceived controllability

Dementia is seen as having severe and several consequences, as indicated by the themes “Dementia as caregiver burden”, “Dementia as stigmatized experience”, “Dementia as transition in relationship dynamics” and “Dementia as enriching experience”.^32,46,49^ Regarding illness coherence, caregivers feel that they lack knowledge and awareness of the disease, which contributes to uncertainty regarding dementia.^31,46,48^ Emotional representations of dementia are identifiable across the themes. Caregivers associate dementia with a variety of negative emotions, such as fear, frustration, being overwhelmed, fatigue, stress, grief, despair, loneliness and isolation,^33,47,55,56^ but experience also positive emotions, e.g., happiness, satisfaction and affection.^32,35,49^

Nevertheless, the model has some limitation in regards to progressive, interactional and dynamic processes of dementia. For example, although the theme: “Transition in relationship dynamics” can be embedded in the self-regulation model as part of the perceived consequences of the disease, this classification is conceptually limited. Changes in personality and relationship dynamics are not merely outcomes of the disease but shape caregivers’ ongoing interpretation of symptoms and behaviors. As the transition from a healthy state to illness is fluid, caregivers can struggle to differentiate between dementia and the person with dementia in their narratives.^19,43^ Thus, symptoms of dementia can be attributed to the personality of the affected person and impact the quality of the relationship. By treating personality and relationship changes as a consequence rather than a dynamic and meaning-laden process, the model underestimates the reciprocal effects of caregivers’ views on dementia and the disease.

To sum up, the self-regulation model might be an overall good fit to capture the caregivers’ views on dementia. Yet, it does not account for the progressive nature of dementia, especially regarding changes in the relationship dynamic.

### Dementia discourse

Views on dementia were diverse, containing positive and negative aspects. With the focus in media shifting to the “living well with dementia” narrative, more emphasis has been put on remaining capabilities and personhood of people with dementia.^70^ This was also captured in the theme: “Dementia as enriching experience”. Although the “living well” narrative can be evaluated as a positive development to destigmatize dementia, it also carries the danger of excluding people with severe dementia symptoms^71^ and puts pressure on caregivers to not discuss taboo topics that contradict the “living well” narrative.^40^ Negative aspects of dementia, especially discussing the sensitive topic “Dementia as transition in relationship dynamics” may be difficult for caregivers. Negative thoughts, beliefs and behavior tendencies regarding people with dementia may not be equated to weakness of character of caregivers or as how they want to view the person with dementia. Instead, they might be seen as consequences of the stress created by the caregiving situation, as highlightened by the theme “Dementia as caregiver burden”. Lack of knowledge, lack of support and stigma may worsen the overall situation for caregivers, as indicated by the themes: “Dementia as uncertainty” and “Dementia as stigmatized experience”. There needs to be a balance between creating a positive public image of people with dementia, while still allowing caregivers to speak up about challenging aspects and receiving the help they need.

### Strengths

To our knowledge, this is the first scoping review on views on dementia among informal caregivers. We created a sensitive search algorithm for multiple databases and included a large number of articles with varying methods and from different countries. Thus, we reduced the chance of missing key studies and selection bias. We combined the scoping review approach with reflexive thematic analysis, i.e., we did not only map the scope of current evidence, but also identified overarching key insights of the heterogeneous papers. Finally, we connected our results to established theories like views on aging and illness perceptions, so that the results were embedded in existing relevant frameworks. In this context, we highlighted similarities and differences leading to a deeper understanding and new perspective on the given constructs.

### Limitations

Certain limitations must be considered when discussing the findings. (a) Although we created a sensitive search algorithm for multiple databases, there is a possibility that we did not identify all the relevant articles, for example due to publication bias, thus leading to limited validity of the results. (b) Furthermore, we were not able to retrieve 26 full texts that might have been eligible for inclusion, as we did not have financial resources to access all journals. We tried to retrieve articles via requests on research gate, but could not obtain all articles this way. (c) As this is a scoping review, we did not provide quality appraisals of the results. We included only peer-reviewed articles from the last ten years to ensure a good level of quality of the papers. Yet, as we did not systematically evaluate them, we cannot guarantee validity, reliability or objectivity of the results of the included studies. (d) One final limitation that should be addressed in future studies is the differentiation of views on dementia based on clinical and sociodemographic variables. Caregivers differ in their situation regarding cultural background, socio-economic status, education, gender, social network, their relationship to and the living situation with the care recipient, the care recipient's type and severity of dementia, etc. These differences might shape experiences and thus views on dementia of caregivers. However, the aim of this review was to find common themes in views on dementia in caregivers worldwide and such analyses would have been beyond the scope of this study.

### Outlook

Results of this scoping review indicate that there is a strong need for conducting further research on views on dementia in caregivers, qualitatively and quantitatively.

Developing and employing a screening tool for views on dementia might help to detect caregivers in critical situations, find compatible support programs and provide therapists or consultants with a better basis to work with their clients, such as providing tailored advice. Furthermore, future research might investigate associations between views on dementia and different health outcomes. Possible outcomes might include physical and mental health burden and help-seeking behavior. In addition, future studies could investigate how views on dementia impact the relationship between caregiver and the person with dementia and consequently, relationship satisfaction and wellbeing of both parts of the caregiving dyad. The present scoping review might inform the development of interventions targeting views on dementia. Further, research on views on dementia could also be targeted towards the general public, i.e., public campaigns to raise awareness and encourage normalization and destigmatization of dementia and a broader discourse about dementia are warranted. This demand for awareness and support programs aligns with WHO's global action plan,^10^ especially as described in the action areas two (Dementia awareness and friendliness) and five (support for dementia carers): The views of caregivers: “Dementia as natural cognitive decline”, “Dementia as stigmatized experience”, “Dementia as self-inflicted vs. externally determined” and “Dementia as uncertainty” point to a lack of knowledge about dementia and a stronger need for a dementia-friendly environment. For example, dementia awareness programs could be established to foster a better understanding of dementia and its subtypes, as well as to reduce stigmatization that is associated with dementia. In particular, the misconception that dementia is a natural part of aging and its associated discrimination might be addressed, as it was a prominent theme in caregivers. The themes “Dementia as caregiver burden” and “Transition in relationship dynamics” emphasize a need for support programs for caregivers in order to equip them with sufficient coping skills and resources to maintain their wellbeing and a good relationship with the care recipient.

Overall, our findings on views on dementia can help to improve the support for caregivers by informing strategies for tailoring support programs for caregivers, reduce misconceptions among caregivers and enabling a broader discourse about caregivers perspective on dementia to reduce stigmatization.
